# Plasma-Induced Oxygen Vacancies in N-Doped Hollow NiCoPBA Nanocages Derived from Prussian Blue Analogue for Efficient OER in Alkaline Media

**DOI:** 10.3390/ijms24119246

**Published:** 2023-05-25

**Authors:** Huu Tuan Le, Ji Eon Lee, So Yeon Yun, Ohyung Kwon, Jin Kuen Park, Young Kyu Jeong

**Affiliations:** 1 Functional Materials & Components R&D Group, Korea Institute of Industrial Technology (KITECH), 137-41 Gwahakdanji-ro, Gangneung-si 25440, Republic of Koreaeon8741@kitech.re.kr (J.E.L.); sy960919@kitech.re.kr (S.Y.Y.); kwonoh@kitech.re.kr (O.K.); 2 Department of Materials Science and Engineering, Korea University, 145 Anam-ro, Seongbuk-gu, Seoul 02841, Republic of Korea; 3 Department of Chemistry, Hankuk University of Foreign Studies, Yongin 17035, Republic of Korea

**Keywords:** oxygen vacancy, plasma etching, N-doped hollow PBA electrodes, PBA-derivation catalyst

## Abstract

Although water splitting is a promising method to produce clean hydrogen energy, it requires efficient and low-cost catalysts for the oxygen evolution reaction (OER). This study focused on plasma treatment’s significance of surface oxygen vacancies in improving OER electrocatalytic activity. For this, we directly grew hollow NiCoPBA nanocages using a Prussian blue analogue (PBA) on nickel foam (NF). The material was treated with N plasma, followed by a thermal reduction process for inducing oxygen vacancies and N doping on the structure of NiCoPBA. These oxygen defects were found to play an essential role as a catalyst center for the OER in enhancing the charge transfer efficiency of NiCoPBA. The N-doped hollow NiCoPBA/NF showed excellent OER performance in an alkaline medium, with a low overpotential of 289 mV at 10 mA cm^−2^ and a high stability for 24 h. The catalyst also outperformed a commercial RuO_2_ (350 mV). We believe that using plasma-induced oxygen vacancies with simultaneous N doping will provide a novel insight into the design of low-priced NiCoPBA electrocatalysts.

## 1. Introduction

There is a need to use clean and renewable energy when people use more energy for economic development. Due to the depletion of fossil fuel resources and increasing environmental pollution, it is important to seek new resources that can be stored, converted, and released on demand. In recent research efforts, scientists have developed various materials for water splitting to provide low-cost catalysts. Electrochemical water splitting could provide hydrogen for fuel cell reactions on demand by using renewable energy such as wind power, hydropower, solar power, bioenergy, etc. However, water electrolysis requires high energy for electron transfer [1]. Noble metal-based materials such as RuO_2_ and IrO_2_ are state-of-the-art resources to develop efficient electrocatalyst electrodes for OERs. Besides good catalyst activity, the drawback of electrodes based on noble metals is the high cost of catalyst material and the availability of noble metals on the Earth. In reality, hydrogen production plants require large electrodes to produce huge amounts of hydrogen. If noble metals are used as catalysts, a large amount will be required, and it is impossible to meet that need because of the high cost and low availability. Those disadvantages could prohibit large-scale applications. The growth of new catalysts is inevitable to reduce costs as well as create environmentally friendly energy sources. Many efforts have been made recently to develop new materials using abundant transition metals to replace noble metals.

The water-splitting reaction comes from two half reactions of oxygen and hydrogen evolution reactions, which require 1.23 V for overall water splitting, known as a kinetic potential for water splitting. Still, most materials need a higher potential for overall water splitting due to many factors, such as the amount of catalyst, exposed active surface area, and conductive solution [2,3]. Many materials have been developed to reduce the overpotential of the water electrolysis reaction. Recently, many transition metals and their derivations have been used to generate new catalysts with low overpotential for OERs, such as Pt_SA_–2D NiHN [4], FeVO(OH)/NiMoW(OH)_2_ [5], CoFeCr LDH [6], and Pt NDs/Cu@Cu_x_O [7]. The bonds from nitrogen–, carbon–, and phosphorous– in catalysts can act as electroactive sites for catalyst activity [8]. The doping technique employed in material science can enhance electrocatalyst performance in HERs and OERs [9]. Other methods are the synthesis of single-atom catalysts for modifying electronic structures at the atomic level to boost the electrocatalyst activity [10,11].

Prussian blue analogues (PBA) are constructed from the coordination of metal ions and cyanide via an in situ cation exchange effect [12,13]. PBA could be prepared using different metal centers (e.g., Fe(CN)_6_^3−^, Fe(CN)_6_^4−^, and Co(CN)_6_^3−^) [14]. PBA could be generated with many synthesis methods using different morphologies and compositions, such as hollow structures [15], nanocages [16,17], flower-like structures [18], and spheres [19]. PBA can be modified through thermal treatment to produce porous metal oxides, selenides, and phosphides depending on the source of inlet gas used. This makes it suitable for many potential applications in electrocatalyst and energy conversion [20]. Recently, PBA-derived materials have been used for water splitting, including CoFe-PBA [21], Co PBA [22], and Co–Cu–Fe PBA [23], with extreme catalysis performance. Therefore, constructing N-doped NiCoPBA-derived self-supported nanocages on nickel foam is a practical strategy for designing efficient oxygen evolution reaction catalysts [24].

Herein, we successfully developed N-doped hollow NiCoPBA nanocages (N_90_–NiCoPBA) using PBA as a structural template on a NF electrode, followed by N_2_ plasma and reduction treatments. PBA template materials can produce catalysts with a large surface area that can improve the OER. Two post processes have been found to play essential roles in enhancing the electrocatalytic efficiency by generating more surface oxygen vacancies and N-doping effects [25,26]. Moreover, direct growth of the PBA sample on NF does not need any polymer binders, which could improve the conductivity and stability of prepared samples in OERs under alkaline environments. This method is a potential way to develop new promising materials for OER applications and overall water splitting in the future.

## 2. Results and Discussion

### 2.1. Structural Characterizations

**Scheme 1:**
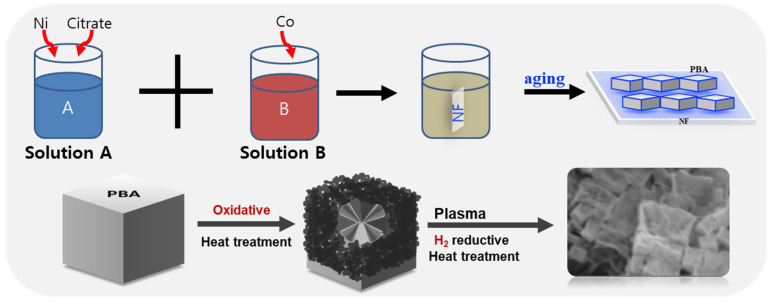
Illustration of N_90_-NiCoPBA synthesis process.

Scheme 1 summarizes the synthesis of N-doped hollow NiCoPBA material by a facial process. Initially, the NiCoPBA was prepared with an aging method to produce a uniformly cubic shape. The benefit of this method produces NiCoPBA at room temperature by using a simple aging method. NiCoPBA was then converted to highly porous H–NiCoPBA by heating at 300 degrees in the air with the benefit of easy evaporation of the –CN bridge from PBA that could provide high-porous materials and improvement in electrolyte diffusion [27]. Nickel ions reacted with free oxygen to form highly crystalline NiO, CoO, and Co_3_O_4_. The N plasma passed through the material surface and reacted with the outer layer to form defects of oxygen and nitrogen doping. The N-doped hollow NiCoPBA was prepared with a highly porous structure that could provide more defections of oxygen, known as oxygen vacancy, for boosting OER catalyst activity.

**Figure 1:**
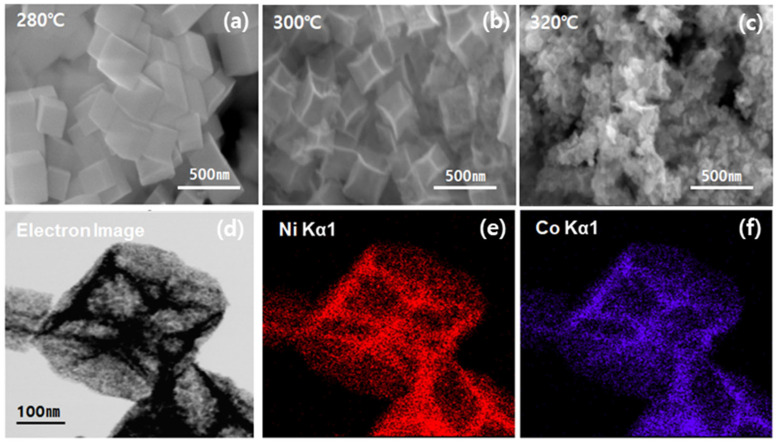
(**a**–**c**) SEM images of H–NiCoPBA after annealing at each temperature for 1 h. (**d**) TEM image of NiCoPBA heat-treated at 300 degrees, and (**e**,**f**) EDS color mapping images representing Ni and Co elements with various colors of N_90_-NiCoPBA.

**Figure 2:**
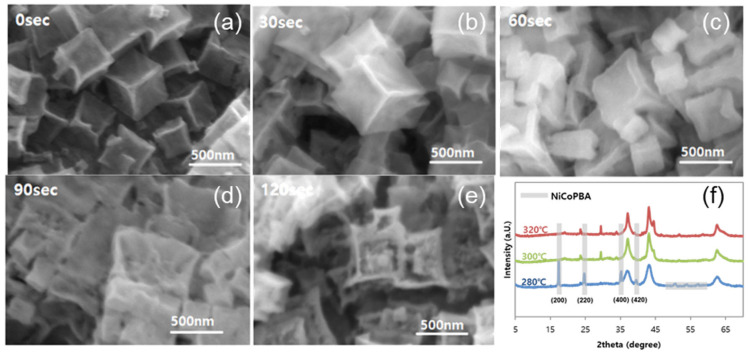
FE-SEM images of N_time_–NiCoPBA with different plasma treatments (time: 0, 30, 60, 90, and 120 s) followed by reduction (**a**–**e**); and (**f**) XRD results of N_90_–NiCoPBA after annealing at different temperatures for 90 s.

The morphology of each material was investigated by the FE-SEM technique. Figure 1a shows H–NiCoPBA annealing at 280 degrees, consisting of highly uniform nanocubes with an average size of ~300 nm. As shown in Figure 1b, the material was converted to a porous structure at 300 degrees without any aggregation. In Figure S1, the H–NiCoPBA shape did not change compared to pristine NiCoPBA, except a porous structure was formed. The material could expose more positions to improve contact between the active site and the electrolyte. Thus, the formation of clusters could reduce the active site of the material, which caused low performance in the OER. The cubic shape was broken and aggregated together, which could turn down catalyst activity (Figure 1c). The material’s microstructure was studied by TEM analysis. The STEM image of the material (Figure 1d) indicated that cubic shape was obtained through simple synthesis by plasma reduction. The presence of Ni and Co elements was confirmed by EDS mapping (Figure 1e,f). As shown in Figure 2, after the N_2_-plasma process, the N-doped hollow NiCoPBA materials showed rough surfaces with distorted cubic morphologies as the processing time increased, implying that the plasma treatment etched more sample structures.

This plasma etching is expected to not only induce surface defects with more active sites, but also incorporate abundant N atoms in NiCoPBA, which is thought to be beneficial for electrocatalytic reactions. The optimized time for plasma treatment was 90 s (N_90_–NiCoPBA) (Figure 2d), which provided the largest etched surface and good catalyst activity compared to other control samples with plasma treatment times of 0, 30, and 60 s (Figure 2a–c). The cubic shape was maintained for 30, 60, and 90 s. However, when the treatment time was increased to 120 s, the morphology became aggregated, leading to a decrease in electrocatalytic performance (Figure 2e). The construction of N_90_–NiCoPBA is optimized when plasma reduction reacts enough with the catalyst to keep a porous structure that does not affect the whole structure.

The structure information of N-doped hollow NiCoPBA was verified by XRD characterization. The crystalline structure can be detected after thermal treatment at 280 degrees with high crystallinity. Peaks at 2-theta of 17, 25, 35.2, and 40 degrees belonged to 200, 220, 440, and 420 planes of pristine PBA material, in agreement with a preview report (JCPDF no. 46–907) [28]. Figure S2 showed the pristine NiCoPBA before the annealing process, with the strong peak located at about 17.3 degrees, which is related to NiCoPBA (JCPDF no. 22–1184) with the chemical formula: Ni_3_[Co(CN)_6_]_2_.3H_2_O [29]. The presence of the CN bridge in the structure of NiCoPBA contributes to the main connection in the crystalline of catalysis. Once this bridge breaks down, the crystal structure of the material will change and, of course, the electronic structure will change as well. This result confirmed that the pristine NiCoPBA has a face-centered cubic structure with high crystallinity. When the temperature increased from 280 to 300 and 320 degrees, all peaks of PBA disappeared (Figure 2f). The disappearance of diffraction peaks was due to the decomposition of PBA at high temperatures to form a highly porous structure of oxide states [30]. Furthermore, products mainly contained oxides of NiO (JCPDS no. 89–3980) and Co_3_O_4_ (JCPDS no. 42–1467), indicating that N-doped hollow NiCoPBA became pyrolyzed from above 300 degrees with a porous structure [31]. The influence of temperature in the crystalline transformation of NiCoPBA can be related to the increase in the oxygen vacancy produced by the reduction in cyanide ligands during the annealing process and the reduction in hydrogen. This escape of cyanide is a significant benefit for making the catalyst become more porous. The porous structure is beneficial for the electrolyte reaching active sites of material to increase the number of electrochemical reactions of catalysts. Exciting, the difference in plasma treatment time is a significant factor while the reduction time is fixed. 

**Figure 3:**
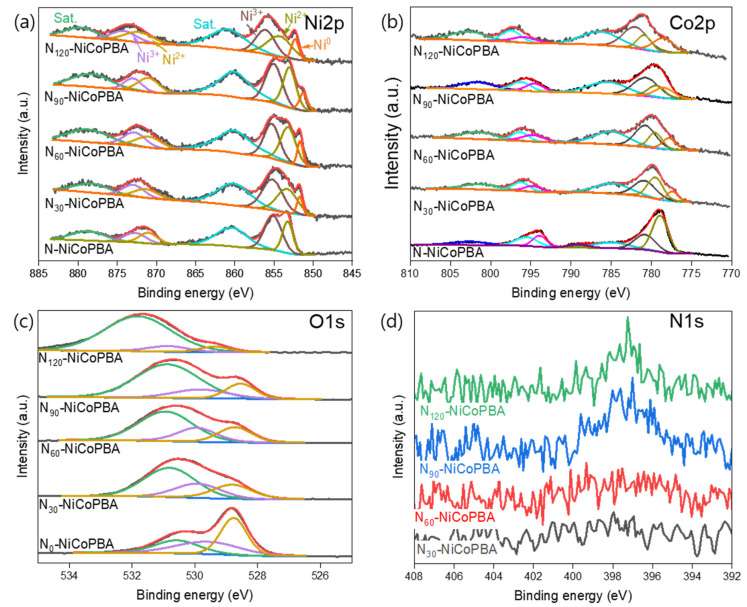
High-resolution XPS spectra of N_0_–NiCoPBA, N_30_–NiCoPBA, N_60_–NiCoPBA, N_90_–NiCoPBA, and N_120_–NiCoPBA for elements of Ni (**a**), Co (**b**), O (**c**), and N (**d**) binding energies.

The electronic structure of N-doped hollow NiCoPBA was studied by XPS spectroscopy. High-resolution peaks of Ni2p of N_90_–NiCoPBA at 851, 853, 855, 871.2, 873.2, and 880 eV belonged to Ni2p3 and Ni2p1/2. Two broad peaks at 860.3 and 878.7 eV were satellite peaks of Ni2p3/2 and Ni2p1/2 [32]. The peak at 851 eV belonged to Ni^0^ (Figure 3a); a clear shift of N_120_–NiCoPBA was confirmed due to defects of oxygen after plasma treatment and followed by reduction. The exposure to the air allows nickel to oxidize to form nickel oxide. This may lead the positive shift to the higher binder energy of Ni2p. The Co2p spectra of N_90_–NiCoPBA (Figure 3b) showed peaks at 778.69, 779.54, 780.8, 794.6, and 796.4, belonging to Co2p3/2 and Co2p1/2 of the Co element (including Co^2+^ and Co^3+^). Two peaks at 785.6 and 801.9 were satellite peaks related to Co^2+^ to Co^3+^ [33,34,35,36]. The phase ratio of Ni^3+^/Ni^2+^ and Co^3+/^Co^2+^ tended to decrease when plasma treatment time increased (Figure S3a,b). The mechanism could be explained as follows: oxygen was removed along with two electrons, which reduced Ni^3+^ to Ni^2+^ and Co^3+^ to Co^2+^. The higher ratio of Co^2+^/Co^3+^ and Ni^3+^/Ni^2+^ from archived catalysts reveals that oxygen vacancy arises from high-temperature annealing under a hydrogen atmosphere [37]. Not only that, Yuanhao et al. made similar Ni/NiFe LDH by activating catalysts using plasma reduction to generate oxygen vacancies in the LDH structure [38]. It is easy to modify the electronic structure of metal hydroxides and oxides of transition metal-based catalysts by using high temperature, introducing plasma, and hydrogen reduction. These results confirmed the existence of oxygen vacancies in the hydroxide structure, which were reported elsewhere [39,40]. The valence state decreased with more oxygen defects (oxygen vacancy), as described previously [41,42]. The spectra of O1s were deconvoluted into three peaks at 528.5, 529.8, and 530.9 eV, corresponding to lattice oxygen in metal oxide and oxygen vacancy (Figure 3c), respectively [43]. As shown in Figure S3c, the phase percentage of oxygen vacancy increased when plasma treatment time increased, indicating that more vacancies were created. The N1s spectra confirmed that the N_90_–NiCoPBA had a broad peak at about 397 eV, belonging to the metal-bound N, which confirmed successful N doping into the NiCoPBA structure [44]. According to Figure 3d, N1s peak positions and intensities were consistent across all samples, suggesting that their electrochemical contributions and chemical state might be equivalent. The benefit of the N-plasma source is that it can generate vacancy oxygen to enhance electrochemical properties and produce nitrogen as a N-doped building [26]. Despite an increase in the abundance of oxygen vacancies with increasing time, an optimal time of 90 s was identified for maintaining the catalyst’s structure and electronic configuration to achieve the best activity. In the catalyst session, we will examine and discuss electrocatalysts using a series of materials that have undergone different plasma treatments.

### 2.2. Catalyst Performance

**Figure 4:**
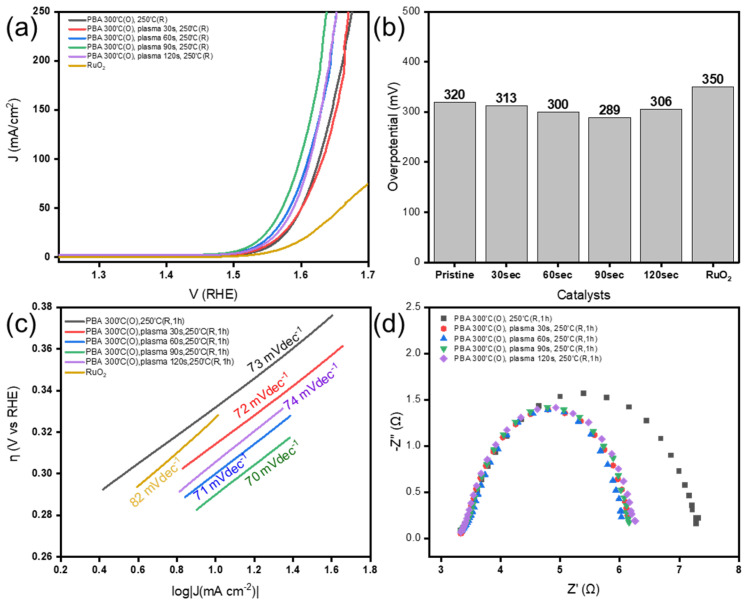
(**a**) LSV measurements of different synthesized catalysts towards OERs in 1 M KOH medium at a scan rate of 5 mV s^−1^; (**b**) Comparison between HER overpotentials of different synthesized catalysts at a current density of 10 mA cm^−2^: N_0_–NiCoPBA, N_30_–NiCoPBA, N_60_–NiCoPBA, N_90_–NiCoPBA, N_120_–NiCoPBA, RuO_2_; (**c**) Tafel slope of material under different plasma treatment times; (**d**) EIS results of N_0_–NiCoPBA, N_30_–NiCoPBA, N_60_–NiCoPBA, N_90_–NiCoPBA, and N_120_–NiCoPBA.

Electrocatalytic properties of the prepared N-doped hollow NiCoPBA electrodes toward OERs were investigated in a three-electrode system under a 1 M KOH solution. As shown in Figure 4a, LSV curves indicated significant differences in OER performance among electrodes. As shown in Figure 4b, the N-doped hollow NiCoPBA had a lower overpotential of 289 mV at 10 mA cm^−2^ than N_0_–NiCoPBA (320 mV), N_30_–NiCoPBA (313 mV), N_60_–NiCoPBA (300 mV), N_120_–NiCoPBA (306 mV), and RuO_2_ (350 mV), indicating the important role of the NiCoPBA structure in OERs. Although RuO_2_ is an active material for OER catalysts, the loading of material on the 3D nickel foam with a lower surface area caused the low catalyst performance compared to NiCoPBA, which was noted by early reports [45,46,47]. Not only that, the performance of RuO_2_ can be associated with charge transfer at the outer surface and inner part, whereas the slow process is due to the charge transfer of deeper and less accessible catalyst layers. Although increasing plasma treatment time could yield more oxygen vacancies, the electrocatalyst activity did not increase. The catalyst’s performance might be reduced because the structure was changed after being exposed to N plasma for a long time, such as 120 s (as discussed in the structural characterization parts). Materials were studied at different annealing temperatures. It was found that 300 degrees was an optimized temperature (Figure S5) with an overpotential of 337 mV, which is much lower than pristine NiCoPBA (Figure S4) with an overpotential of 403 mV. These results indicate the electrocatalyst activity was enhanced when the structure was modified. The improvement of the electrocatalyst is related to the escape of the CN group and defects in the crystal structure. The reaction centers make easy contact with the solution to exchange electrons for generating oxygen from OH^−^. While the catalyst structure is maintained after plasma reduction for 90 s, the catalyst treated in 120 s affected the entire structure, resulting in submaximal reaction efficiency, which is discussed in the XRD session. The reduction process enhanced the electrocatalyst activity by reducing the overpotential value from 337 mV (H–NiCoPBA) to 289 mV (N_90_–NiCoPBA), which is higher than 2.5 Fe–NiCoP [48], Co_3_O_4_/NiO@CeO_2_ [49], SLa–Ni_x_Co_1–x_(OH)_2_ [50], Ni–Co PBA cage [31]. These results showed that using the N-plasma source for 90 s could produce a hollow structure with a high surface area. Treatment in the plasma source followed by thermal reduction provided more oxygen vacancies, generating extra unpaired free electrons on the material’s surface. The primary mechanism was that the outer layer reacted with N plasma to allow N doping into hollow NiCoPBA with highly exposed surface-active sites. The OER reaction process could be explained as follows [4]:

(1) $$Me+OH^{-}\to MeOH+e^{-}$$

(2) $$MeOH+OH^{-}\to MeO+H_{2}O+e^{-}$$

(3) $$MeO+OH^{-}\to MeOOH+e^{-}$$

(4) $$MeOOH+OH^{-}\to Me+O_{2}+H_{2}O+e^{-}$$

**Figure 5:**
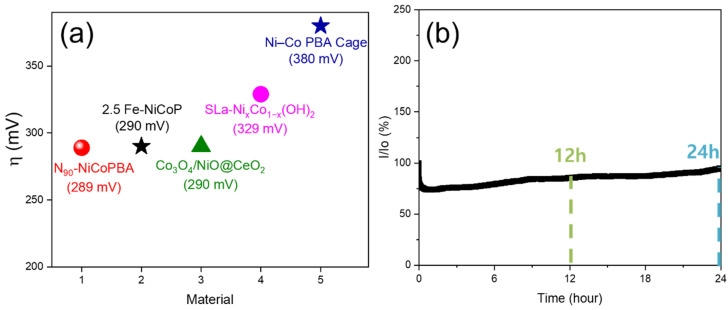
(**a**) Comparison of overpotential of N_90_–NiCoPBA catalyst with recent works at a current density of 10 mA cm^−2^, (**b**) Chronoamperometric measurements of N_90_–NiCoPBA material towards long-term OER operation.

To study long-term stability of the N_90_–NiCoPBA catalyst, chronoamperometry was used to investigate OER performance. The material showed superior stability at a potential of 1.54 V for 24 h. The current density of the material was maintained at 84.7% for the first 12 h and 95.1% after 24 h of operation (Figure 5b). These results indicate that N-doped hollow NiCoPBA is stable in an alkaline medium for a long time, which can enhance performance and reduce cost.

## 3. Materials and Methods

### 3.1. Materials

Nickel (II) nitrate hexahydrate (Ni(NO_3_)_2_·6H_2_O, 99.9%), sodium citrate (C_6_H_5_Na_3_O_7_·2H_2_O, ≥99%), and potassium hexacyanocobaltate (III) (K_3_Co(CN)_6_, ≥97.0%), ruthenium (V) oxide (RuO_2_ ≥ 99%), ethanol (C_2_H_5_OH, 95%), and acetone (CH_3_COCH_3_, 99.9%) were purchased from Sigma-Aldrich. Nafion™ 117 containing solution (Nafion 5%) was purchased from Sigma-Aldrich Chemicals Co. (St. Louis, MO, USA). Nickel foam was purchased from Changsha Lyrun Material Co., Ltd (Changsha, China).

### 3.2. Synthesis of NiCoPBA/NF

A piece of nickel foam (NF) (0.5 cm × 3 cm) was cleaned with 3M HCl solution (15 mL) and acetone (15 mL) for 30 min using ultrasonication. The NF was then washed several times using ethanol and distilled water.

In a typical synthesis, Ni(NO_3_)_2_·6H_2_O (0.6 mM) and sodium citrate dihydrate (0.9 mM) were dissolved in 20 mL of deionized (DI) water to form solution A. K_3_[Co(CN)_6_] (0.4 mmol) was dissolved in 20 mL of DI water to form solution B. Then, solutions A and B were mixed by stirring for 1 h to make a homogenous solution C. The pretreated nickel foam was immersed into Solution C and aged for 7 days at room temperature. Afterward, the NF was cleaned with ethanol and water and dried at 70 °C in a vacuum oven. The dried NiCoPBA/NF was then dipped in solution C again and aged for 6 days before undergoing washing and drying processes.

### 3.3. Synthesis of Hollow NiCoPBA/NF (H–NiCoPBA/NF)

The NiCoPBA/NF was put on a ceramic boat and heated at 300 °C in an electric oven for 2 h in the air to convert NiCoPBA/NF to hollow NiCoPBA/NF (H–NiCoPBA/NF). 

### 3.4. Synthesis of N-Doped Hollow NiCoPBA (N_90_–NiCoPBA/NF)

The N_90_–NiCoPBA/NF was prepared using the following process. Typically, the sample was attached to a wafer using ketone tape. Plasma treatment was then performed with the conditions: source power: 1000 W; pressure: 10 mT; bias power: 500 W; and gas flow of N_2_: 150 sccm and Ar: 30 sccm for 90 s. After plasma treatment, the sample was heated in a hydrogen atmosphere using a tube furnace. Each specimen was placed at the center of a tube furnace and calcined at 250 °C with H_2_ and Ar flow rates of 100 and 100 sccm for 1 h to convert H–NiCoPBA/NF to N_90_-NiCoPBA/NF. Other control samples were prepared with different plasma treatment times of 0, 30, 60, and 120 s for comparative studies of OER activity.

### 3.5. Material Characterizations

Field-emission scanning electron microscopy (FE-SEM) was used to analyze the morphology of each material on a Quanta 250 FEG (FEI Company, Hillsboro, OR, USA) at the Korea Institute of Industrial Technology (KITECH, Gangwon Division, Korea). The structure of each material was studied by transmission electron microscopy (TEM) using a Talos F200X microscope (Thermo Fisher Scientific, Waltham, MA, USA) equipped with an EDX analyzer at 200 kV at the Korea Basic Science Institute Wonju Center at Gangwon National University. The crystallinity of each material was determined by X-ray diffraction (XRD) on a D/Max 2500 V/PC (Rigaku Co., Tokyo, Japan) with a Cu target (λ = 0.154 nm) at the KITECH. The surface chemistry of each material was investigated by X-ray photoelectron spectroscopy (XPS) on a VG ESCALAB 200i instrument (Thermo Fisher Scientific) at the Gangwon National University Center for University-wide Research Facilities (CURF). Ar–N_2_ inductively coupled plasma (ICP) used 2300 Versys KIYO Poly (LAM RESEARCH, Fremont, CA, USA) at KITECH. Process conditions were as follows: pressure, 10 mT; source power, 1000 W; bias power, 500 W; gas flow, N_2_–150 sccm; and Ar, 30 sccm.

### 3.6. Electrochemical Measurements

An Autolab workstation (Metrohm Autolab B.V. Netherlands at KITECH Gangwon Division, Korea) connected with a three-electrode cell was used to investigate electrochemical properties of materials. A N90–NiCoPBA/NF electrode (0.5 × 0.5 cm) was used as a working electrode, and a platter of platinum and Ag/AgCl was applied as counter and reference electrodes. Materials were examined by linear sweep voltammetry (LSV) with a scan rate of 0.5 mV s^−1^ to record the polarization curve of different materials towards OERs in a 1 M KOH medium. Stability and durability of electrodes towards OERs were investigated using the amperometry technique. Charge transfer resistance of electrodes was measured by electrochemical impedance spectroscopy (EIS) in a frequency range (10^5^ to 10^−2^) Hz. For comparison, commercial RuO_2_ powder was also used to prepare electrodes by dispersing 0.5 mg of catalyst (similar to loading N_90_–NiCoPBA) into a 0.5 mL solution of a 20 µL binder polymer (Nafion 5%) and 0.5 mL ethanol under sonication for 20 min to form a suspension. The solution was slowly loaded onto the NF substrate to achieve an electrode for OER measurements. 

## 4. Conclusions

A hollow NiCoPBA material was assumed straightforwardly. It showed excellent catalyst performance. The optimized time for plasma treatment was 90 s, which provided more oxygen vacancies for OER catalysts. The electrocatalytic performance of the N-doped hollow NiCoPBA depended on the number of oxygen vacancies. Under optimized conditions, N90–NiCoPBA showed remarkable OER performance with an overpotential of 289 mV and a Tafel slope of 70 mV/dec. The N-doped hollow NiCoPBA also demonstrated excellent durability in an alkaline medium for more than 24 h at 10 mA/cm2. Improved electrocatalytic performance was due to oxygen vacancies in N-doped hollow NiCoPBA nanocages, which offered great active sites and a 3D porous substrate that enhanced electrical conductivity. This study highlights successful preparation and optimization of N-doped hollow NiCoPBA for efficient water splitting.

## Data Availability

Not applicable.

## Supplementary Materials

The following Supporting Information can be downloaded at: https://www.mdpi.com/article/10.3390/ijms24119246/s1.
