# Supplementary material for: Plasma-Induced Oxygen Vacancies in N-Doped Hollow NiCoPBA Nanocages Derived from Prussian Blue Analogue for Efficient OER in Alkaline Media
Source: Int J Mol Sci. 2023 May 25;24(11):9246. doi: 10.3390/ijms24119246 (PMC10252958; doi:10.3390/ijms24119246)
Supplement: Supplementary file 1 [file ijms-24-09246-s001.zip › ijms-2393268-supplementary.pdf]

Supporting information

Plasma-Induced Oxygen Vacancies in N-doped Hollow NiCoPBA Nanocages Derived from Prussian Blue Analogue for Efficient OER in Alkaline Media

Huu Tuan Le[1,†], Ji Eon Lee[1,2,†], So Yeon Yun[1,3], Ohyung Kwon[1], Jin Kuen Park[3,*], Young Kyu Jeong[1,*]

[1] Functional Materials & Components R&D group, Korea Institute of Industrial Technology (KITECH), 137–41 Gwahakdanji-ro, Gangneung-si, Gangwon 25440, Republic of Korea.

[2] Department of Materials Science and Engineering, Korea University, 145 Anam-ro, Seongbuk-gu, Seoul 02841, Republic of Korea.

[3] Department of Chemistry, Hankuk University of Foreign Studies, Yongin, 17035, Gyeonggi-do, Republic of Korea

[†] These authors contributed equally to this work.

[*]E-mail: jinkpark@hufs.ac.kr, immrc@kitech.re.kr

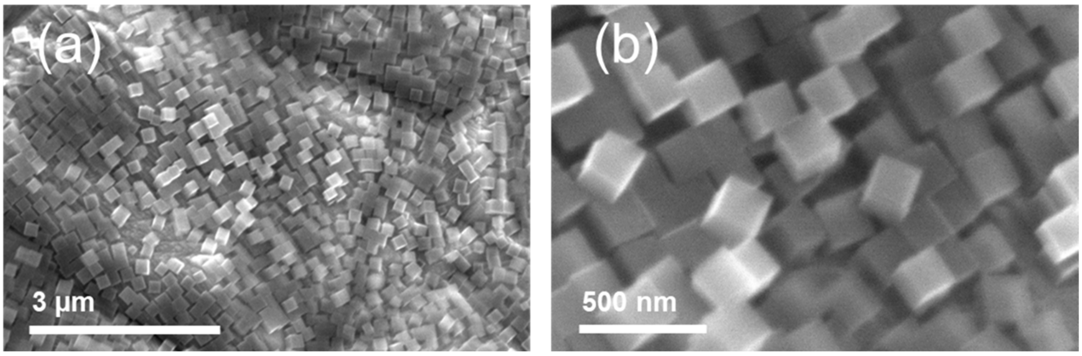

Figure S1. SEM image of Pristine NiCoPBA and high magnification. (a) 3 μm, (b) 500 nm

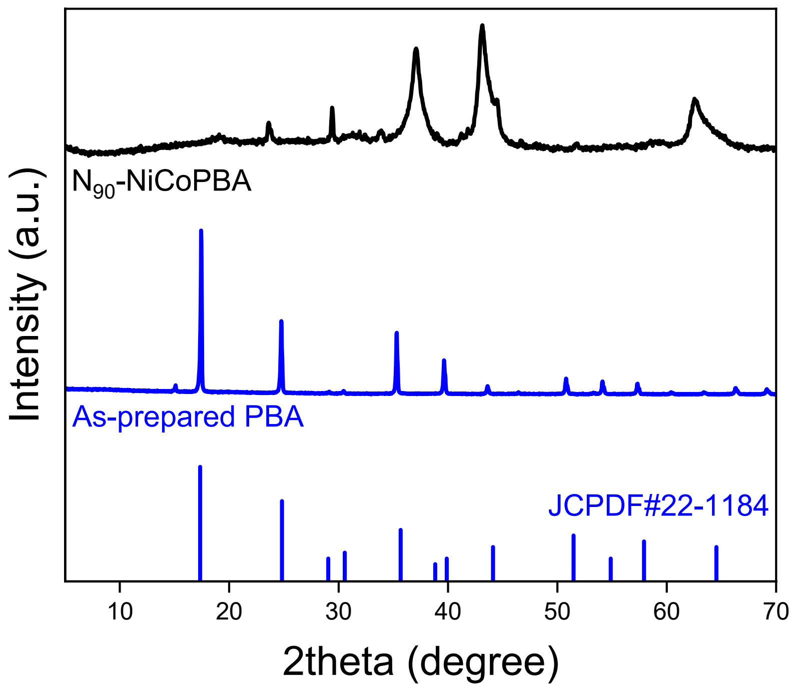

Figure S2. XRD results of Pristine NiCoPBA and N_{90}-NiCoPBA.

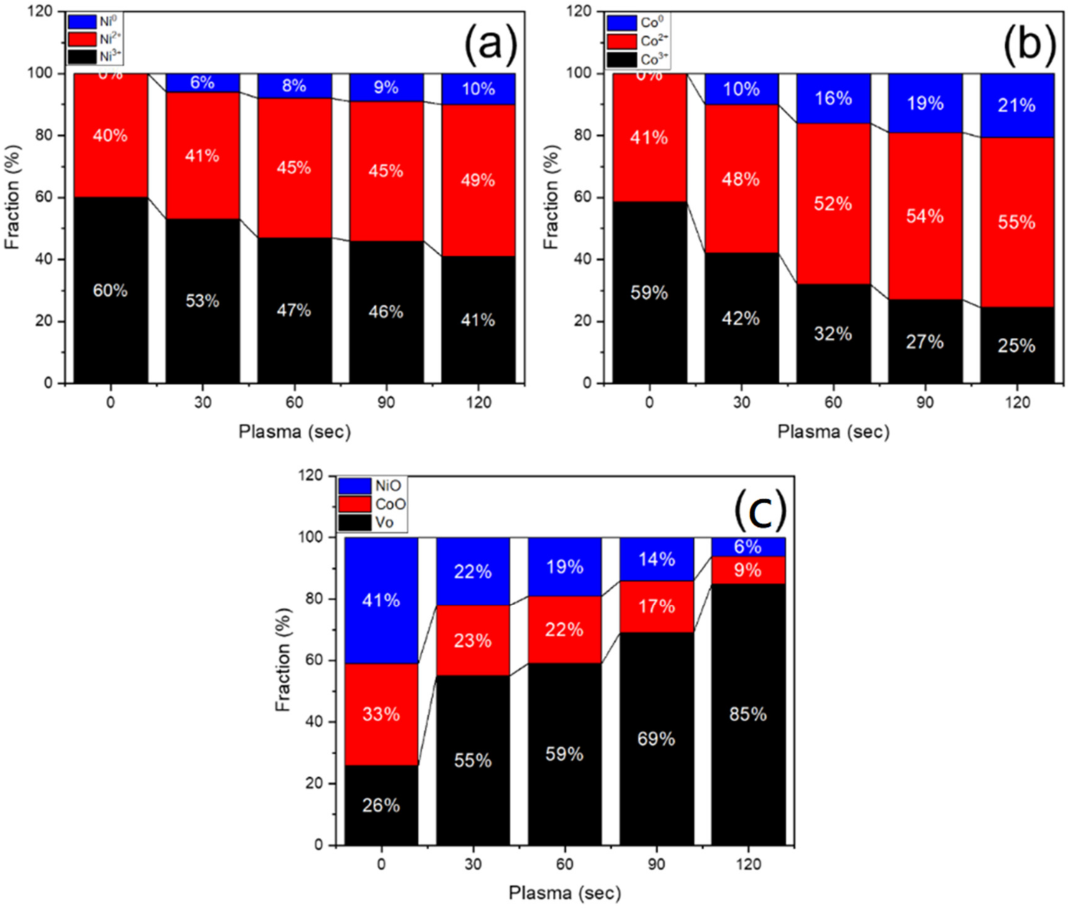

Figure S3. Phase percentage of (a) Ni2p, (b) Co2p, (c) O1s from deconvolution of high resolution XPS.

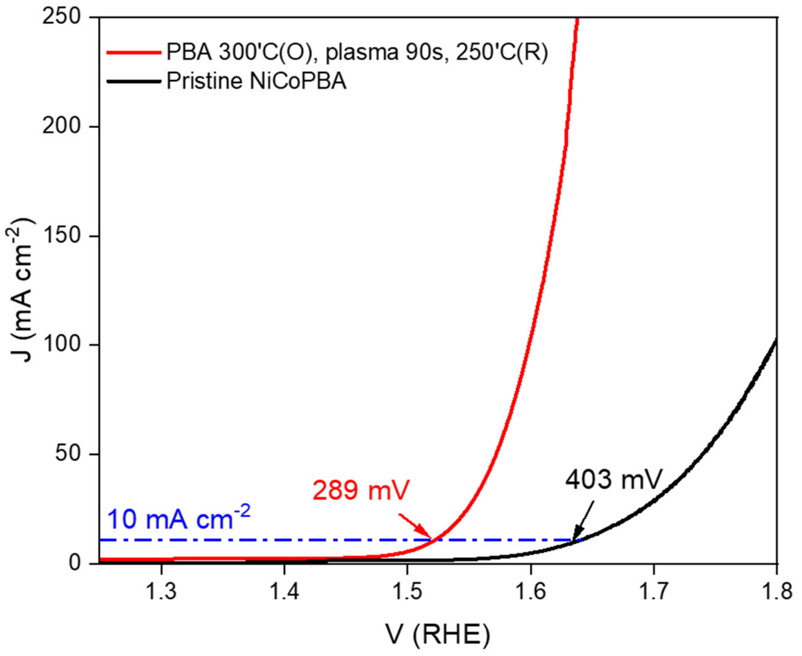

Figure S4. Linear sweep voltammetry (LSV) curves of Pristine-PBA and N_{90}-NiCoPBA at a scan rate of 0.5 mV s^{-1}.

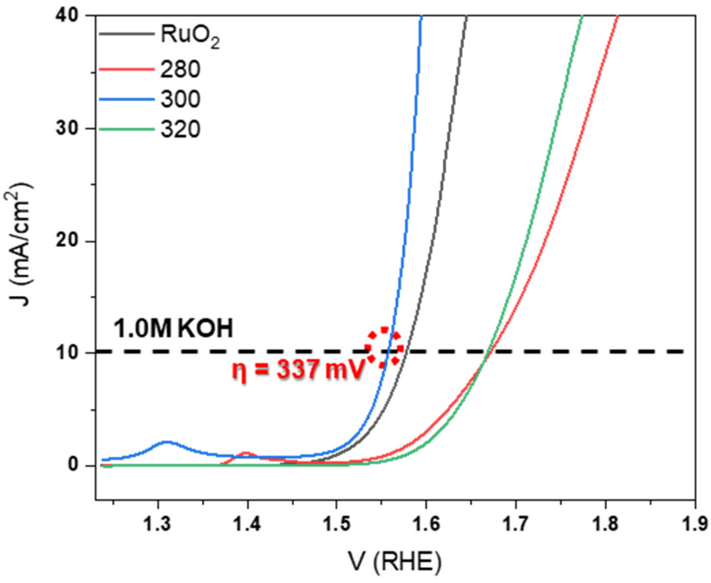

Figure S5. Linear sweep voltammetry (LSV) curves were recorded at a scan rate of 0.5 mV s^{-1}.
